# Inter-Individual Variability of Stone Marten Behavioral Responses to a Highway

**DOI:** 10.1371/journal.pone.0103544

**Published:** 2014-07-29

**Authors:** Fernando Ascensão, Clara Grilo, Scott LaPoint, Jeff Tracey, Anthony P. Clevenger, Margarida Santos-Reis

**Affiliations:** 1 Centro de Biologia Ambiental, Faculdade de Ciências da Universidade de Lisboa, Lisboa, Portugal; 2 Western Transportation Institute, Montana State University, Bozeman, Montana, United States of America; 3 Departamento de Biologia & CESAM, Universidade de Aveiro, Aveiro, Portugal; 4 Max-Planck-Institute for Ornithology, Radolfzell, Germany; 5 Department of Biology, University of Konstanz, Konstanz, Germany; 6 US Geological Survey, Western Ecological Research Center, San Diego, California, United States of America; University of Delhi, India

## Abstract

Efforts to reduce the negative impacts of roads on wildlife may be hindered if individuals within the population vary widely in their responses to roads and mitigation strategies ignore this variability. This knowledge is particularly important for medium-sized carnivores as they are vulnerable to road mortality, while also known to use available road passages (e.g., drainage culverts) for safely crossing highways. Our goal in this study was to assess whether this apparently contradictory pattern of high road-kill numbers associated with a regular use of road passages is attributable to the variation in behavioral responses toward the highway between individuals. We investigated the responses of seven radio-tracked stone martens (*Martes foina*) to a highway by measuring their utilization distribution, response turning angles and highway crossing patterns. We compared the observed responses to simulated movement parameterized by the observed space use and movement characteristics of each individual, but naïve to the presence of the highway. Our results suggested that martens demonstrate a diversity of responses to the highway, including attraction, indifference, or avoidance. Martens also varied in their highway crossing patterns, with some crossing repeatedly at the same location (often coincident with highway passages). We suspect that the response variability derives from the individual's familiarity of the landscape, including their awareness of highway passage locations. Because of these variable yet potentially attributable responses, we support the use of exclusionary fencing to guide transient (e.g., dispersers) individuals to existing passages to reduce the road-kill risk.

## Introduction

The negative impacts of roads on wildlife have long been recognized [1]–[5]. Among their many impacts, roads may act as physical barriers to moving animals, thereby reducing landscape connectivity [6], [7]. This barrier effect is augmented when wildlife-vehicle collisions (WVC) become significant mortality sources for populations [8], [9]. Both WVC and the barrier effect have numerous fitness consequences (e.g., reduced gene flow) that can severely reduce long-term population viability [10], [11].

Measures to reduce WVC and mitigate the barrier effect are diverse [12] but wildlife fences combined with crossing structures are gaining more attention by road agencies as they prevent animals from accessing roads while maintaining the connectivity between roadsides [12]–[14]. However, the choice of mitigation strategy to apply often relies on general patterns, for example road-kill clusters or movement responses [15]–[17], on the basis that these patterns provide information on the average response of species to roads and traffic. Hence, if individuals vary widely in their responses to roads or mitigation actions and mitigation efforts are directed toward population-level average responses, these efforts may be only partially effective [18]–[20].

The life stage and state of an individual can affect its behavioral response to both the road and to the mitigation actions, such as transient individuals avoiding interactions with residents [7], [21], [22]. For example, squirrel glider (*Petaurus norfolcensis*) movements were re-established across a highway after canopy bridges and glider poles were installed [18], [23], yet only half of the individuals known to be present in the vicinity of a canopy bridge used the bridge [18]. The authors suggest that by actively defending their territories, and the passages within them, resident gliders exclude others from accessing those passages [18]. Such behavior could influence monitoring survey results, leading to spurious conclusions on mitigation effectiveness. The importance of thoroughly understanding individual response variability to roads and mitigation is clear.

This knowledge is particularly important for medium-sized carnivores that are especially vulnerable to the negative impacts of roads [8], [24], [25]. These species typically travel great distances to maintain their territories and occur at low population densities [26], [27]. These traits increase their probability of encountering roads and the significance of each negative interaction. Further, because of their size, these species are often able to trespass through road exclusionary fences, thus being highly exposed to road-kill risk [28]–[31]. However, it has been shown that these species also regularly use available road passages (e.g., drainage culverts) for safely crossing highways [32]–[34].

Our goal in this study was to assess whether this apparently contradictory pattern of high road-kill numbers associated with a regular use of road passages is attributable to the variation in behavioral responses toward the highway between individuals. We reanalyzed the tracking data of seven stone martens (*Martes foina*, hereafter referred to as ‘marten’) previously described by [15]. To our knowledge, this is the only available carnivore tracking dataset from a study area that also contains data on road-kill [28] and passage use [33] patterns. As carnivores often occur at low densities, studies investigating their responses to roads often suffer from low sample sizes [35], [36], precluding the application of robust analytical methods [37]–[39]. To overcome small sample size limitations, we employed a novel analytical framework that compares the observed utilization distribution, response turning angles, and highway crossing patterns to results from simulations parameterized with observed data for each individual. We considered these response patterns to describe distinct levels of road impact on marten movements: a greater impact is expected if the utilization distribution across the home range is affected by the highway; an intermediate impact when turning angles are affected by highway proximity; and a more localized impact is expected if crossing patterns are affected by road passage location. Our study design provides a rigorous analytical framework to investigate individual behavior that can be applied across many species and landscapes making it of interest to ecologists, conservation biologists and road planners seeking to understand and mitigate the impacts of roads on carnivore populations.

## Materials and Methods

### Study area

**Figure 1:**
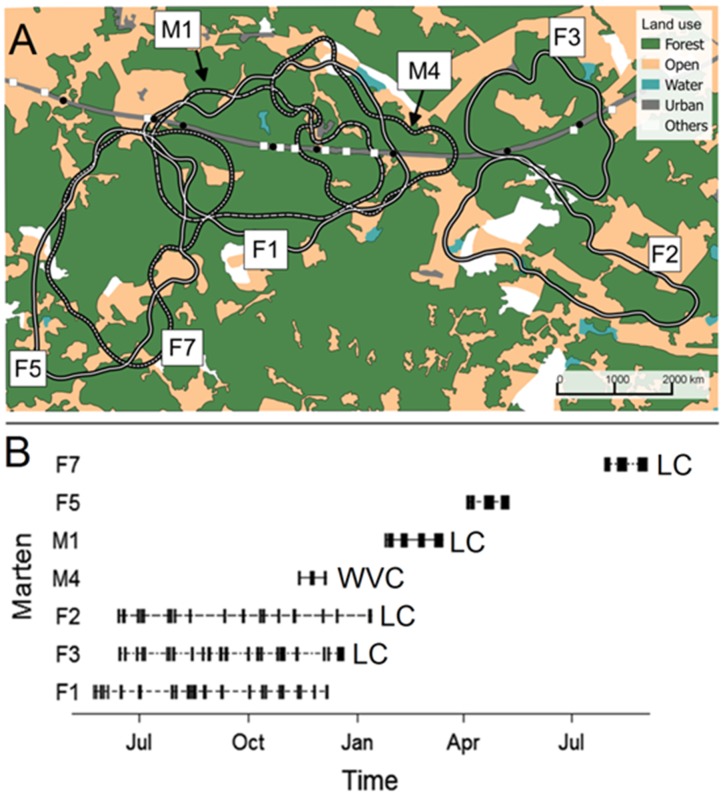
A: Highway A6 in southern Portugal and its crossing structures (squares - culverts, circles - under/over passages), land covers, and marten home range areas (white lines). B: Duration (2008–2009) of tracking nights for each marten (each bar is one night) with “LC” indicating loss of contact and “WVC” indicating a confirmed WVC (corpse recovered). Apparent home range overlap of F1 with M1 and M4, and F5 with F7 correspond to distinct periods.

Martens were tracked in the Mediterranean region of southern Portugal (39°38.154′N, 8°12.128′W), an area dominated by cork-oak woodlands (*Quercus suber*). The study area includes an approximately 10 km section of the four-lane A6 highway and its adjacent surroundings (Fig. 1A). This highway was built in 1995 and has a speed limit of 120 km/h. During the martens activity period in this region (i.e., 20:00 to 08:00 [40]), the A6 receives 169+/−159 vehicles/hour (BRISA S.A., highway enterprise). This highway section has 21 crossing structures available to martens: 13 culverts (1.0–1.5 m in diameter) for water flow, seven underpasses (5 m high, 8 m wide) and one overpass, both for cars and agricultural machinery.

### Study species and dataset

The stone marten is a medium-sized carnivore occurring across parts of Asia and Europe [41]. It is often tolerant of human settlements [42], but in our study area it is more commonly associated with cork-oak woodlands [40], [43]–[45]. They are typically solitary and territorial, with home ranges reaching 2 to 3 km^2^ [40]. These martens are particularly sensitive to forest fragmentation [40], [43], [44], [46], and are also highly vulnerable to road-kills, being the second most frequently road-killed carnivore in southern Portugal [28].

We selected seven individuals (two males and five females) that had sufficient data from the dataset used by [15] (Table 1, individual identification herein is that of [15]). These individuals were tracked between April 2008 and September 2009 during 136 tracking nights (mean 19+/−7 per marten). Each night, one marten was intensively tracked by two observers who attempted to locate the marten every 30 minutes (see [15] for more details). This effort yielded 1425 locations (mean 10+/−5 locations per night per marten) with a mean time between successive locations of 39+/−22 min (Table 1). Pairwise comparisons using Wilcoxon rank sum test with Bonferroni correction revealed a significant higher time interval between relocations in F3 than F2 (p = 0.008). However, given the small difference (ca. 5 min.), we do not expect this to preclude the behavior comparison between martens.

**Table 1:** Summary data for each marten considered in the present study.

Marten	Number of tracking sessions	Tracking hours	Number of fixes	Mean time between relocations (min)
F1	28	122	202	51±22
F3	28	190	300	44±44
M1	19	137	238	39±22
M4	5	36	64	37±13
F2	19	118	213	39±13
F5	19	124	205	41±16
F7	18	124	203	43±21

Tracked time and time between relocations includes only tracking sessions with at least two successful relocations. Individuals are sorted by whether they crossed the highway (F1, F3, M1, and M4) or not (F2, F5, and F7).

### Data analysis

#### Utilization distribution

We estimated marten utilization distributions (UD) with biased random bridges (BRB), a movement-based kernel density estimation method [47], [48]. This method improves the spatial resolution of UD estimates by considering activity times between serially correlated relocations rather than simply the spatial density of these relocations as if they were unlinked. The BRB model inserts interpolated locations at regular intervals between each observed location and then uses classical kernel estimation, with a variable smoothing parameter dependent on the time between successive relocations, to estimate the UD [48]. Rather than requiring independence between successive locations, as other space use estimators require [49], [50], the BRB model uses the time between successive locations to parameterize the biased random walks between each location [48]. Thus, as the time between successive locations decreases, the width of the bridges (i.e., the size of the area within which the individual may have passed through between successive fixes) decreases, thereby producing a more realistic probability of the animal's true path. Marten space use was estimated within their home range area, which we defined as the 95% isopleth of their UD. BRB were calculated using the ‘BRB’ function within the ‘adehabitatHR’ package (version 0.4.2) [51], [52] for R [53].

#### Movement response angles

We used the nonlinear regression model described by [54] to model the response angles of martens when they approached the highway. The parameters of this model allow us to infer the qualitative response of martens (i.e., attraction, avoidance, or indifference) to the highway proximity. Response angles (*A_i_*) are defined as the difference between the angle of direction to the highway at step *S*_i_ and the angle of direction at *S*_i+1_,where a step is the estimated path of the animal between successive locations (Fig. S1). These models use the von Mises distribution that is characterized by both the mean angle (μ, angle of maximum probability density) and a concentration parameter (*k*) that controls the dispersion of the distribution about the mean angle, analogous to the precision of a normal distribution [54]. The distribution is symmetric about the mean angle μ. When *k* = 0, the distribution is uniform on -π to π radians. As *k* increases, the distribution concentrates about the mean angle [54]. We considered two nonlinear models, hereafter referred to as the ‘no-response’ and ‘responsive’ models.

In these models, μ is set constant (i.e., independent of the animal-to-object distance, *T*_i_). In the ‘no-response’ model, *k* is also independent of the proximity of the object, in this case the highway. Conversely, for the ‘responsive’ model, the concentration parameter is dependent on the animals distance to the highway: the strength of the animal's response is expected to increase with decreasing distance to the highway, so that the animal has a greater tendency to move in the mean response angle μ. As the distance between the animal and the highway increases, the von Mises distribution becomes uniform and the animal's movement direction becomes more independent of its distance to the highway.

In the ‘responsive’ model the decay of *k* with the distance to the highway follows an exponential function, governed by two parameters, θ_1_ and θ_2_. These two parameters measure the concentration of the von Mises distribution when the distance to the object is zero (θ_1_) and the rate of decay of the strength of the animal's response as it gets farther from the object (θ_2_). Statistical inference is likelihood-based, similar to those for generalized linear models to obtain the maximum likelihood estimates [54]. If the highway does not influence marten movement, the ‘no-response’ model will best fit the observed data, whereas the ‘responsive’ model should best fit martens that respond strongly (either attraction or avoidance) to the highway. We excluded locations of inactive martens for this analysis.

#### Highway crossing patterns

We identified highway crossings as pairs of consecutive marten locations during the same tracking session recorded on opposite sides of the highway. For each marten, we counted the number of crossings and calculated the utilization distribution using these pairs of locations (UD_cross_) also with the BRB method, similar to the approach used by [55], [56].

#### Null model procedures

We used a null model approach to determine the influence of the highway on marten utilization distribution and highway crossing patterns. Note that the models used to analyze the response angles already incorporate a comparison with a ‘no-response’ model. Null models are pattern-generating simulation models that deliberately exclude a mechanism of interest (for our purposes, the presence of a highway), and by using randomization procedures allow the user to test the importance of that mechanism in observed patterns [57]–[60]. To build a null model, first an observation is recorded (e.g., number of crossings) from which a set of simulations guided by a set of randomization rules is generated and the simulated response is measured. A large number of iterations (e.g., 1000) are used to generate a frequency histogram of expected response values. The position of the observed response within this null distribution indicates the probability value of the observed pattern, just as in a conventional statistical analysis [58].

Simulated movements were parameterized with the attributes of the observed data (i.e., the number of tracking sessions, locations, step lengths, and utilization distribution boundary), but the simulated agent was naïve to the presence of the highway. For each tracking session, an agent (i.e., simulated marten) started from an observed resting site, chosen randomly, and then moved the same number of steps whose length followed the observed step lengths' sequence. The agents' successive location must fall within the home range boundary at a random direction from the previous location. Therefore, simulations follow a constrained random walk which has been successfully used in previous road ecology studies [61]–[64]. This process is repeated for each tracking session of each marten and each simulated location is saved for further analysis.

For each response considered - utilization distribution, frequency and location of highway crossings - we performed a set of 1000 simulations, per marten. Each set of simulations was used to generate a frequency distribution, from which the confidence intervals of the observed response were estimated. Based on likelihood significance tests, we considered an effect of the highway if the observed parameter fell outside the 5–95% percentiles of the simulated parameter distributions. The model was built using NetLogo 4.1.3 [65] and is available as Model S1.

#### Influence of land cover on marten movement

Prior to analysis, we investigated marten habitat selection in the study area using a weighted compositional analysis as described by [66]. We obtained the land cover information by directly classifying Google Earth images using the ‘OpenLayers’ plugin in QGIS (version 1.8) [67]. Ground observations were used to check for and correct potential mismatches. All patches of forest (cork-oak, 67% of the area), agricultural (crop or fallow, 27%), urban (2%), water bodies and streams (1%) present in our study area were polygonized in GIS. Remaining areas were classified as “other” (3%) (Fig. 1A). These land cover polygons were then rasterized to a 30 m grid using the ‘raster’ package (version 2.0-41) [68] for R [53].

For each marten home range, we calculated the sum of the probability values of all cells of the UD for the land cover classes ‘forest’ and ‘agricultural’, and considered these proportions to be the ‘available’ habitat. The ‘used’ habitat was estimated by calculating the proportion of locations that fell within forest or open per marten. The test was performed using the command ‘compana’ in the R package ‘adehabitatHS’(version 2.15.1) [51] with a randomization test (1000 permutations).

## Results

### Influence of land cover on marten movement

We found no evidence for marten habitat selection (Λ = 0.72, p = 0.18) and so excluded land cover information from further analyses. This was not unexpected, as stone martens in the region are not forest-specialists [40].

### Utilization distribution

**Figure 2:**
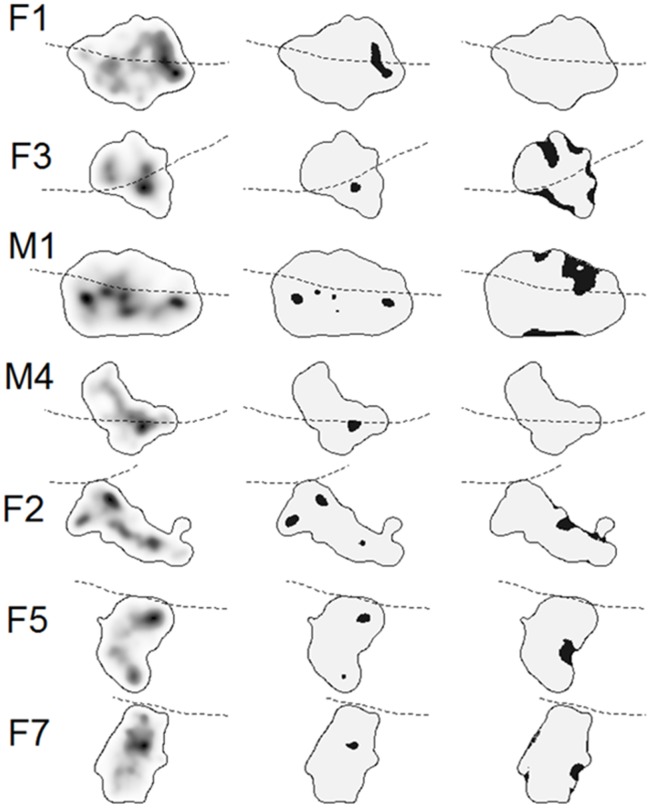
Left column: the utilization distributions (UDs, from biased random bridges) of tracked martens with increasing shading indicating increasing use intensity. Marten home-ranges were computed as the 95% isopleth of the UDs. Middle and right columns: black areas suggest areas where martens spent more and less time than expected by chance (95% or 5% of simulations), respectively. The highway is shown in each plot by the dotted line. Images are scaled (among martens). Individuals are sorted by whether they crossed the highway (F1, F3, M1 and M4) or not (F2, F5 and F7).

The UDs of martens whose home ranges overlapped with the highway (F1, F3, M1 and M4) revealed inconsistent patterns of space use near the highway (Fig. 2, left column), with some areas near the highway being used more than expected (Fig. 2, middle column). Less used areas were generally located near territory boundaries and, except for M1, with no relation to highway proximity (Fig. 2, right column).

### Movement response angles

Marten responses to the highway were highly variable between individuals. The response angles of five of the seven martens were best predicted by the ‘responsive’ model, suggesting most martens showed a significant response to the highway (Table 2). For example, the mean response angle (μ) of F1 suggested an attraction toward the highway (μ near zero), although the concentration of the von Mises distribution when the distance to the highway approach zero (θ_1_) was low (Table 2). Territories of martens F2, F5 and F7 did not overlap with the highway and consequently their mean response angles were high. However, θ_1_ was also variable among them, denoting different avoidance levels to the highway proximity. For example, F2 and F5 had θ_1_ values of 2.55 and 10.75, respectively, suggesting that the latter had a stronger response to move away from the highway proximity (Table 2). Marten M4 had a mean angle of 1.61, with a θ_1_ relatively high, suggesting a predominantly movement parallel to the highway when in its proximity. Interestingly, results for two martens crossing the highway (F3, M1) suggest that their movement was not influenced by highway proximity. The decay of the response angle (θ_2_) was low for all martens whose movement was best explained by the ‘responsive’ model, suggesting a nearly linear relation of θ_1_ with distance to the highway (Table 2).

**Table 2:** Maximum likelihood estimates of parameters for the ‘responsive’ model: μ - mean angle (in absolute values, ranging from 0 to |π| radians); θ_1_ – strength of concentration parameter when marten is at distance zero from the highway; θ_2_ – rate (exponential) of decay of the concentration parameter as the animal moves farther the highway.

Marten	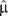	θ_1_	θ_2_	?^2^
F1	0.24	0.33	0.000	8.7 (0.00)
F3	1.16	1.32	0.045	2.6 (0.11)
M1	2.30	0.84	0.013	2.3 (0.13)
M4	1.61	1.09	0.008	5.6 (0.02)
F2	2.96	2.55	0.004	6.3 (0.01)
F5	3.08	10.75	0.004	9.3 (0.00)
F7	2.62	6.66	0.005	15.1 (0.00)

Last column stands for the comparison of movement responses to highway proximity, where ‘no-response’ and ‘responsive’ nonlinear models are compared by likelihood ratio test (degrees of freedom = 1). Between brackets is the p-value for the test. Individuals are sorted by whether they crossed the highway (F1, F3, M1, and M4) or not (F2, F5, and F7).

### Highway crossing patterns

**Figure 3:**
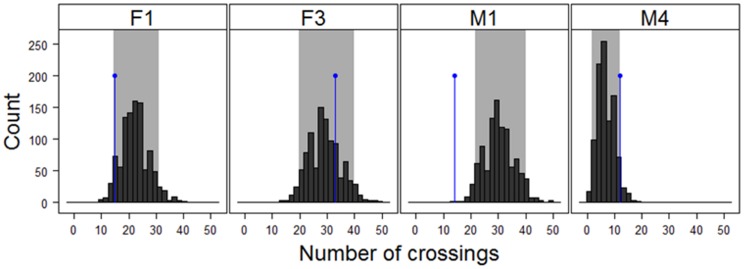
Histograms showing the simulated (i.e., predicted) frequency of marten highway crossings (grey bars) and the observed number of crossings (black dot). Grey areas represent the percentile (5–95%) envelope of reference from the simulated datasets. Dots outside of the percentiles suggest the individual crossed less often (left) or more often (right) than expected.

**Figure 4:**
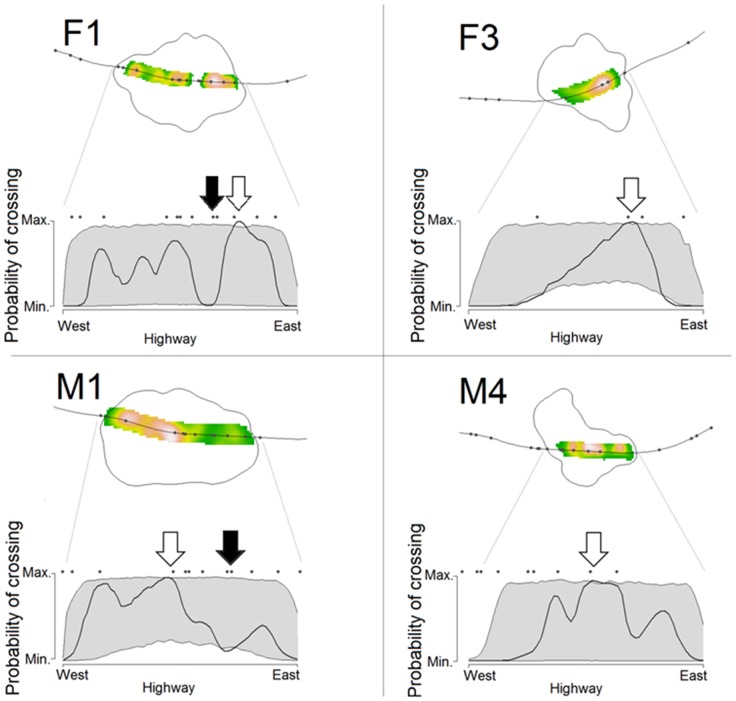
Per marten, top: the utilization distribution of marten highway crossing locations within 200 m from the highway (UD_cross_). Bottom: the observed probability of crossing the highway at each road segment (UD_cross_ at highway location; solid line). Grey areas represent the 5–95% percentile envelope of reference from the simulated datasets. White (black) arrows indicate highway segments with higher (lower) use than expected. Points indicate road passage location. For each marten, the highway segment in the upper-half of the figure is projected in the X axis from the bottom picture.

Marten crossing patterns varied between individuals, suggesting no general pattern in highway crossing frequency or crossing locations (Fig. 3). Marten M1 and marginally F1 crossed the highway less often than expected, while M4 crossed more often than expected and F3 crossed as often as expected (Fig. 3). For these four martens, their UD_cross_ values suggested that they tended to cross near highway passages. Although most of their UD_cross_ were within the expected interval from the simulations, the highway segments that were used more often than expected have passages (white arrows in Fig. 4). This was particularly clear for F1 and F3. The exception was M4, for which some crossings apparently occurred in sections without any passages (Fig. 4). Interestingly, both F1 and M1 seemed to avoid crossing the highway where paved roads pass beneath the highway (black arrows in Fig. 4), suggesting a possible behavioral avoidance of this passage type (Fig. 4).

## Discussion

Our individual-based analytical framework improved our understanding of how martens respond to the presence of a highway. All martens demonstrated some level of influence of the highway proximity for each of the behavioral responses we considered. However, their responses were more variable than expected [15], highlighting the complexity of individual behavioral toward these linear structures. Because we found no clear evidence for marten habitat selection, we believe that these behavior patterns were mainly due to individual responses to the presence of the highway and crossing structures therein.

We were able to provided new insights into the apparently contradictory results of previous work held in same study area, where martens were frequently killed on the highway [28] while also regularly using crossing structures [33]. We hypothesize that this apparent contradiction could stem from differences between individuals in their familiarity of the highway and the location of passages. For example, seasonal peaks in road mortalities have been well documented elsewhere and coincide with seasonal behavioral patterns, such as breeding behavior, provisioning young or spring dispersal events [28], [69], [70]. Presumably, these peaks occur because dispersing individuals or individuals exploring new areas in search of mates may be unaware of the passage locations and naïvely cross over the highway, increasing their mortality risk. The support for our hypothesis is described below.

As previously described by [15], some martens maintained territories that overlapped the highway while others maintained territories adjacent but not overlapping with the highway. However, our results suggest that martens from the former group spent more time than expected in some areas near the highway, while others apparently had no influence of highway proximity on their space use. Linear infrastructures have been used as home range boundaries by carnivores [7] and the martens may have been hunting within the highway verge where prey densities are high [71], although martens with territories adjacent to the highway apparently did not. Hence, we suspect that this high use of the highway verge is due to searching for and using highway crossing structures. This is supported by as the response angles of two martens seem uninfluenced by the highway, which suggests that the highway represented no deterrent for their movement, and they used crossing locations coincident with existing highway crossing structures. Nevertheless, despite the knowledge that these martens regularly cross roads [15], their crossing rates appear highly variable. Moreover, M4 moved parallel to the highway when in its proximity, being the only individual that crossed the highway more often than expected, at both locations coincident and without crossing structures.

Overall, although the sample size of our dataset is less than ideal, we show that martens can exhibit a variety of responses to the highway, especially in their propensity to cross the highway and to use crossing structures to do so. We assume that martens F1, F2 and F3 were residents with well-established territories since they were tracked for long periods, having stable home range areas [15], and F1 and F3 were apparently aware of passage locations for crossings. The movement of these two martens was not hindered by the presence of the highway probably because they knew where to access suitable crossing structures. Although we did not monitor the existing road passages, *ad-hoc* observations confirmed that F3 regularly used the structure with higher probability of use (author's *pers. obs.*, passage bellow the white arrow in Fig. 4).

We also believe that M4 was dispersing through the region as he was not detected before being captured, despite the continuous and intense trapping effort [15]. This marten was trapped in November when martens typically disperse [72], [73] and was road-killed shortly after arriving to the study area. Assuming this marten was not a resident he would have been naïve to the location of passages or could have been prevented from accessing them. This would explain that, unlike other martens, some crossings of M4 occurred in sections without passages. Our assumption that non-residents are unaware of the passage locations for safe crossings is supported by previous work suggesting that individuals require time to adapt to existing crossing structures [74]–[77]. Thus, our findings suggest that the use of passages seems to be governed not only by road and environmental attributes [78], but also by individual preferences and familiarity with the landscape.

To effectively mitigate the negative effects of roads at the population level we must understand the processes that affect the movements of individuals and the variability between individual responses to roads and existing mitigation [16]. For example, marten use of crossing structures, particularly culverts, is well documented [32]–[34], [79], [80]. However, our results suggest individual preferences for specific crossing structures: F3 crossed the highway at least 30 times during our tracking sessions, but apparently did so through a single passage despite at least two similar structures being within her home range (Fig. 4). Additionally, F1 and M1 both used and avoided the same passages (white and black arrows, respectively, in Fig. 4). These differences may reflect individual preferences for passage characteristics, locations, or both.

An important research question remains: how many individuals use the passages? If only a few individuals regularly use the same passage, as our results suggest, then the effectiveness of these structures could be overestimated [18]. This topic is of major importance [12], yet poorly understood [but see 18], [81]. This information, together with a deeper understanding of animal behavior near roads, would provide a spatio-temporal bridge between the individual and its population [82], [83]. Mitigation strategies that ignore this information may be insufficient. Given the amount of time these martens spend near the highway, the variety of responses they demonstrated toward the highway, and their apparent passage-type preferences, we believe mitigation strategies would be more effective by optimizing the number of and spacing between passages [84], and by directing new individuals toward existing passages via exclusionary fencing with a sufficiently small mesh size as previously suggested [85], [86]. Such mitigation measures are necessary to reduce the mortality risks to both resident and importantly to dispersing individuals.

## Supporting Information

Figure S1 **Diagram illustrating the response angle in relation to the highway location (grey line).** The animal moves from S_i_ to S_i+1_. The animal-to-highway angle in radians is C_i_. The move angle is B_i_ and the response angle is A_i_.
(DOCX)

Model S1 **NetLogo model built to simulate marten movement in highway vicinity.**
(NLOGO)
