# Supplementary material for: Inter-Individual Variability of Stone Marten Behavioral Responses to a Highway
Source: PLoS One. 2014 Jul 29;9(7):e103544. doi: 10.1371/journal.pone.0103544 (PMC4114789; doi:10.1371/journal.pone.0103544)
Supplement: Figure S1 — Diagram illustrating the response angle in relation to the highway location (grey line). The animal moves from Si to Si+1. The animal-to-highway angle in radians is Ci. The move angle is Bi and the response angle is Ai. (DOCX) [file pone.0103544.s001.docx]

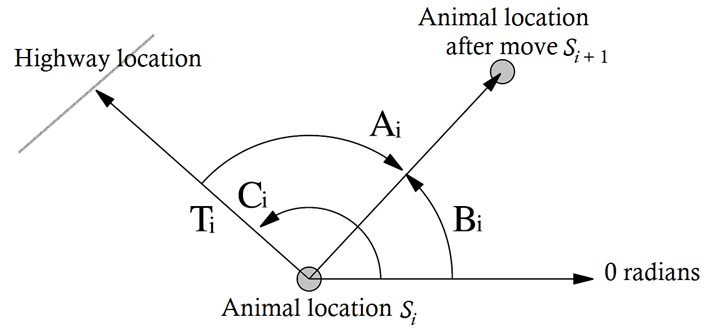


Figure S1 – Diagram illustrating the response angle in relation to the highway location (grey line). The animal moves from S_i_ to S_i+1_. The animal-to-highway angle in radians is C_i_. The move angle is B_i_ and the response angle is A_i_. Adapted from Tracey *et al.* (2005).
